# Pain Reactivity and Plasma β-Endorphin in Children and Adolescents with Autistic Disorder

**DOI:** 10.1371/journal.pone.0005289

**Published:** 2009-08-26

**Authors:** Sylvie Tordjman, George M. Anderson, Michel Botbol, Sylvie Brailly-Tabard, Fernando Perez-Diaz, Rozenn Graignic, Michèle Carlier, Gérard Schmit, Anne-Catherine Rolland, Olivier Bonnot, Séverine Trabado, Pierre Roubertoux, Guillaume Bronsard

**Affiliations:** 1 Laboratoire Psychologie de la Perception, Université Paris Descartes, UMR 8158 CNRS, Paris, France; 2 Service Hospitalo-Universitaire de Psychiatrie de l'Enfant et de l'Adolescent de Rennes, Université de Rennes 1, Rennes, France; 3 Child Study Center, Yale University School of Medicine, New Haven, Connecticut, United States of America; 4 Assistance Publique-Hôpitaux de Paris, CHU Bicêtre, Service de Génétique moléculaire, Pharmacogénétique et Hormonologie, Le Kremlin-Bicêtre, et INSERM U 693, Université Paris-Sud 11, Faculté de Médecine Paris-Sud, Kremlin-Bicêtre, France; 5 Centre Emotion, UFR 3246 CNRS, Groupe Hospitalier Pitié-Salpétrière, Paris, France; 6 Laboratoire de Psychologie Cognitive, UMR 6146 CNRS, Université de Provence, Aix en Provence, France; 7 Centre Hospitalier Universitaire de Reims, Reims, France; 8 Centre référent maladies rares à expression psychotique, Service de Psychiatrie de l'Enfant et de l'Adolescent, Groupe Hospitalier Pitié-Salpétrière, Paris, France; 9 INSERM U 910, Génétique Médicale Génomique Fonctionnelle, Université Aix-Marseille 2, Marseille, France; 10 Maison des Adolescents et Centre Médico-Psycho-Pédagogique des Bouches-du-Rhônes, Laboratoire de Santé Publique (EA3279) à la Faculté de Médecine de Timone, Marseille, France; Deutsches Krebsforschungszentrum, Germany

## Abstract

**Background:**

Reports of reduced pain sensitivity in autism have prompted opioid theories of autism and have practical care ramifications. Our objective was to examine behavioral and physiological pain responses, plasma β-endorphin levels and their relationship in a large group of individuals with autism.

**Methodology/Principal Findings:**

The study was conducted on 73 children and adolescents with autism and 115 normal individuals matched for age, sex and pubertal stage. Behavioral pain reactivity of individuals with autism was assessed in three observational situations (parents at home, two caregivers at day-care, a nurse and child psychiatrist during blood drawing), and compared to controls during venepuncture. Plasma β-endorphin concentrations were measured by radioimmunoassay. A high proportion of individuals with autism displayed absent or reduced behavioral pain reactivity at home (68.6%), at day-care (34.2%) and during venepuncture (55.6%). Despite their high rate of absent behavioral pain reactivity during venepuncture (41.3 vs. 8.7% of controls, *P*<0.0001), individuals with autism displayed a significantly increased heart rate in response to venepuncture (*P*<0.05). Moreover, this response (Δ heart rate) was significantly greater than for controls (mean±SEM; 6.4±2.5 vs. 1.3±0.8 beats/min, *P*<0.05). Plasma β-endorphin levels were higher in the autistic group (*P*<0.001) and were positively associated with autism severity (*P*<0.001) and heart rate before or after venepuncture (*P*<0.05), but not with behavioral pain reactivity.

**Conclusions/Significance:**

The greater heart rate response to venepuncture and the elevated plasma β-endorphin found in individuals with autism reflect enhanced physiological and biological stress responses that are dissociated from observable emotional and behavioral reactions. The results suggest strongly that prior reports of reduced pain sensitivity in autism are related to a different mode of pain expression rather than to an insensitivity or endogenous analgesia, and do not support opioid theories of autism. Clinical care practice and hypotheses regarding underlying mechanisms need to assume that children with autism are sensitive to pain.

## Introduction

It has often been stated that individuals with autistic disorder have reduced pain sensitivity. Parents, caregivers and mental health professionals have reported that some autistic children appear to withstand painful stimuli (bumps, cuts, etc.), show absence of nociceptive reflexes (e.g., absence of hand withdrawal reflex when burning oneself), or absence of guarded body position in cases of broken legs or arms. However, nearly all of the support for this idea is derived from anecdotal reports and limited clinical observations [1]–[9]. Despite the paucity of systematic studies of pain sensitivity and reactivity in autism, the presence of pain insensitivity in autism has been given added credence through its inclusion as an associated feature in standard diagnostic texts. In DSM-IV and DSM-IV-TR “a high threshold for pain” is mentioned [10], [11] while in DSM-III the ignoring of pain is described [12].

A number of researchers have suggested that excessive brain opioid activity could explain the apparent pain insensitivity of autism and contribute to or even determine the pathogenesis of autism [4]–[9]. The opioid theories in autism have been based on 1) symptom profiles seen in autism, 2) reported therapeutic effects of opiate antagonists and 3) reported abnormalities in opioid levels measured in individuals with autistic disorder, such as β-endorphin (BE). An increase of central BE activity in autism would be consistent with a purported reduced pain sensitivity and elevated pain threshold [1], [2]–[4]. There are also apparent symptom similarities between autism and opiate addiction or behavioral states following administration of opiate and opioid agents in animals [13]–[19]. Autistic children and opiate-addicted organisms both appear less sensitive to pain, less emotional and asocial.

Additionally, autistic children show stereotyped and self-injurious behaviors (SIB) which have been suggested to be related to pain insensitivity and to be mediated by abnormally high levels of BE [20]–[23]. It should be noted that opioid peptides interact with numerous opioid receptor types [23], [24], and that BE itself acts with the similar affinity at mu, delta and epsilon receptors. The non-selective opiate antagonists naltrexone and naloxone have been reported to have beneficial effects on autistic symptoms including SIB, stereotypies, social withdrawal and productive speech [20], [21], [23]. However, benefits have not been consistently demonstrated [24], [25] and one study [25] found that naltrexone actually increased stereotypies in autism.

As shown in **Table 1**, a large number of studies have measured levels of plasma BE in autism without reaching a consensus. The contradictory data concerning plasma BE levels in autism could be partly explained by methodological problems, i.e. specificity of immunoassays, small sample size and clinical heterogeneity of autism. Studies of central opioid levels in autism have also produced inconsistent results [37], [41], with CSF BE levels in autistics reported to be increased, decreased or similar to controls (see **Table 1**). In a study of the relationship between decreased pain sensitivity and CSF endorphin, mean CSF endorphin fraction II levels (mainly Met-enkephalin) were reported to be elevated in autism spectrum disorder [37]. While this study raised important issues regarding the relationships between analgesia and endorphin dependency in autism, pain reactivity was assessed as a dichotomous variable without distinguishing different clinical forms of pain reactivity.

**Table 1:** Studies of Endorphin and Enkephalin Levels in Individuals with Autism.

Study	Fluid	Autism Group (N)	Opioid	Results
Weizman et al. 1984 [26]	Plasma	10	Humeral endorphin	Decreased compared to normals
Herman et al. 1986 [27]	Plasma	5	BE	Similar to normals
Weizman et al. 1988 [28]	Plasma	22	BE	Decreased compared to normals or schizophrenics
Bouvard et al. 1992 [29]	Plasma	4	BE	Increased compared to normals
Ernst et al. 1993 [30]	Plasma	5	BE	Similar to normals
Leboyer et al. 1994 [31]	Plasma	67	N-terminal BE	Decreased compared to normals or girls with Rett's
Leboyer et al. 1999 [32]	Plasma	62	C-terminal BE	Increased compared to normals or girls with Rett's
Bouvard et al. 1995 [33]	Plasma	10	C-terminal BE	Increased compared to normativecontrol values
Tordjman et al. 1997 [34]	Plasma	48	BE	Increased compared to normals
Brambilla et al. 1997 [35]	PBMC*	12	BE	Increased compared to normals or PDD
Cazullo et al. 1999 [36]	PBMC	11	C-terminal BE	Increased compared to normals
Gillberg et al. 1985 [37]	CSF**	20	Met-enkephalin	Increased in SIB and low pain sensitivity
Ross et al. 1987 [38]	CSF	9	BE	Increased compared to normals
Gillberg et al. 1990 [39]	CSF	31	BE	Decreased compared to normal adults
Nagamitsu et al. 1993 [40], 1997 [41]	CSF	19	BE	Similar to normals

* PBMC indicates peripheral blood mononuclear cells.

** CSF indicates cerebrospinal fluid.

The study of pain sensitivity and reactivity in autism is inherently difficult, but accumulating parallel studies of pain in children [42]–[44] in neurological impairment [45], [46] and psychiatric disorders [47], and in mental retardation and developmental delay [48]–[51] provide a framework for investigation. In general, the more recent studies have underscored the influence of setting, the importance of having multiple raters, the difficulty in assessing the absolute and perceived magnitude of the painful stimulus, and the problems presented by limited or unusual behavioural repertoire when attempting to assess pain sensitivity.

We have carried out the present study in order to clarify issues of pain reactivity and sensitivity in individuals with autistic disorder, to examine possible alterations in plasma BE in autism, and to investigate the potential associations of BE with behavioral and physiological responses to pain. Behavioral pain reactivity was examined in children and adolescent with autistic disorder using extensive behavioral assessments in different observational situations. Physiological response to pain was assessed by measuring heart rate changes during venepuncture, while possible alterations in BE and its potential mediating effects were examined after measurement of plasma BE levels in large groups of subjects and across severity subgroups of autistic disorder. The measurement of plasma BE was undertaken in order to better understand the potential role of the opioid system in autistic behaviors and pain-related behaviors in autism. Greater understanding of the underlying mechanisms could have important implications regarding the treatment and care of individuals with autism. Children and adolescents were studied due to the inherently greater relevance of measurement made in younger subjects when studying an early onset developmental disorder, and due to relevant prior published reports examining children and adolescents with autism. Plasma BE was measured due to the invasiveness of cerebrospinal fluid sampling, because of the lack of demonstrated utility of urine BE measurements, and due to the existence of several prior reports of plasma BE concentrations in autism.

This study was part of a larger project that had as its overall objective the examination of associations between behavioral profiles and biological variables in children with autism. The larger project included, in addition to the study of plasma BE and pain-related behaviors, an examination of the relationship between aggression and plasma testosterone concentrations and between social withdrawal and platelet serotonin levels.

## Methods

### Subjects

Children and adolescents with autistic disorder (n = 73) were recruited from child day care facilities of the University Hospital of Bicêtre (Faculté de Médecine de Paris-Sud, recruitment by S. Tordjman M.D., Ph.D.) and of the University Hospital of Reims (recruitment by M. Botbol M.D., AC Rolland M.D., G Schmit M.D. and Ph.D.)) and were group matched with normal control individuals (n = 115) on the basis of sex, age and Tanner stage of puberty as assessed by pediatricians [52]. Demographic data are given in **Table 2**.

**Table 2:** Demographic Characteristics of Study Groups.

Variable	Autism Comparison Group Group	(n = 73) (n = 115)	*df* Test Statistic (*P* values>0.10)
Sex M/F	49/24	75/40	1 χ^2^ = 0.02
Pubertal status* (Pre-pubertal/Pubertal/Post-pubertal)	32/16/25	45/27/43	2 χ^2^ = 0.32
Age, Mean±SD, (years)			
Total group	11.7±4.5	12.7±5.9	186 *t* = 1.37
Males	11.7±4.5	13.1±6.4	122 *t* = 1.31
Females	11.6±4.5	12.3±5.5	62 *t* = 0.63
Pre-pubertal	8.1±2.7	7.2±2.2	75 *t* = 1.61
Pubertal	13.8±3.4	12.5±1.8	41 *t* = 1.64
Post-pubertal	16.9±3.7	18.5±4.6	66 *t* = 1.60

* Pre-pubertal = Tanner stage 1; Pubertal = Tanner stages 2, 3 and 4; Post-pubertal = Tanner 5.

Based on direct clinical observation by two independent child psychiatrists, a diagnosis of autistic disorder was made according to DSM-IV [11], ICD-10, and CFTMEA [54] criteria, and was confirmed by the Autism Diagnostic Interview-Revised (ADI-R) [54]. Normal controls, recruited from the preventive medical center of Reims where they went for a regular check-up, were determined by two independent paediatricians to be free of significant psychopathology and any developmental or neurological disorders. Additionally, there was no family history of autism in the control group. All subjects were Caucasian, physically healthy and had no history of encephalopathy or neuroendocrinological disease. All control individuals were unmedicated, while 47 of the patients with autism were unmedicated. Of the 26 medicated patients with autism, 14 patients had a history of idiopathic epilepsy and were being treated with anticonvulsants, and 15 patients were receiving neuroleptics. The research protocol was approved by the ethics committee of Bicêtre Hospital and written informed consent was obtained from parents.

### Behavioral and Cognitive Assessments

Cognitive functioning of individuals with autistic disorder was assessed using the age-appropriate Wechsler intelligence scales (WPPSI-R, WISC-R, WAIS-R) and the Kaufman K-ABC [55]. All patients were cognitively impaired: mean full scale IQ = 42.2, SD = 3.2 (range 40–58).

Behavioral assessments were performed using the ADI-R [54] and the Pre-Linguistic Behavioral Pain Reactivity Scale (PL-BPRS) [56]. Autism severity was assessed using the ADI-R, leading to an overall score of impairments for the combined Social, Communication and Stereotypy domain (ranging from 1 to 3; the ‘0’ coding means “not autistic”), using a methodology previously described [57]. Based on ADI-R rating, 52 of the 73 patients with autistic disorder were identified to be non-verbal. The overall rating of autistic impairments for each patient was then used to dichotomize subjects according to autism severity. Individuals were grouped into mild-moderate (score 1–2) and severe (score 3) impairment. Inter-judge reliability with respect to the critical distinction between mild/moderate and severe impairment was excellent, with an inter-judge agreement of 95% observed between the two expert raters. The PL-BPRS assessed behavioral pain reactivity by rating the patients' apparent and observable reactions to noxious stimuli. This scale is qualitative, categorizing behavioral pain reactivity in 5 classes summarized in **Table 3**. Additionally, the type of accidental painful stimulus (burn, painful illness, cut, pinch, etc.) was noted. Painful stimuli provoked by SIB were not considered. The PL-BPRS has been previously found to be reliable and valid for assessment of pain reactivity in autism [56]–[58].

**Table 3:** Types of Pain Reactivity assessed by the PL-BPRS (Pre-Linguistic Behavioral Pain Reactivity Scale).

**Type I: Paradoxical pain reactivity**
Apparent pleasure reaction to a painful stimulus (such as smiling or laughing).
**Type II: Absence of pain reactivity**
Absence of any reactions described in class IV with absence of nociceptive reflexes (such as absence of hand withdrawal reflex when burning oneself or absence of arm withdrawal reflex from the needle during a blood drawing).
**Type III: Hyporeactivity to pain**
The individual with autism appears to withstand pain, but nociceptive reflexes to nociceptive stimuli are present. After noxious stimuli, the following possible abnormalities are observed: incomplete pain reactivity compared to class IV, abnormally delayed reaction time.
**Type IV: Normal pain reactivity**
After painful stimuli the following reactions are observed: cries, screams, moaning, grimaces, reflexes of nociceptive withdrawal, lack of movement, body orientation and glance towards the painful area (e.g., glance towards the venepuncture or sometimes away from it, also the individual with autism takes the observers hand to put it on the painful area of his/her body, a behavior often found in some non-verbal patients with autism), stopping an activity in progress, reaction when the injured area is touched, guarded body position when resting or in movement, spontaneous protection of the painful area. One of the important signs of pain reactivity for individuals without verbal language is the immobilisation of the painful area of the body.
**Type V: Hyperreactivity to pain**
Disproportionate cries and screams given the painful stimulus (with hypersensitive light touch).

Pain reactivity was assessed for patients in three different observational situations. 1) in day-care, where two caregivers independently rated overall pain reactivity on a daily basis during the month preceding the blood drawing; 2) at home, where parents rated pain-related behavior during the same month as the caregivers. In this situation, there were enough daily life situations involving pain to distinguish reactions to a variety of types of noxious and painful stimuli such as being burned, having internal pain (tooth pain, ear infection, headache, etc.), and other accidental painful stimuli (cutting, pinching, banging, etc.); 3) during the blood drawing at a medical center, when a direct clinical observation was conducted by a nurse and child psychiatrist not belonging to the caregiver team. Normal controls were similarly assessed for pain reactivity to the venepuncture using the PL-BPRS. During the blood drawing, heart rate was measured (with a stethoscope placed on the thorax considering that some patients can react negatively when their wrist is touched) immediately before and after the venepuncture (15-second measurement period) in order to assess cardiovascular response to the needle insertion. In addition, a checklist was used to indicate the presence or absence of SIB, aggressive behaviors directed against others, stereotyped behaviors and social withdrawal during the blood drawing situation.

### Blood drawing procedures and biochemical analyses

The blood drawing for patients with autism (n = 63) occurred at the nearest general hospital rather than at the day-care facility so that the research procedure was not associated with the therapeutic milieu. Blood drawing for controls (n = 115) occurred at the Preventive Medical Center. The blood drawing followed a standardized procedure to minimize and control the possible stressful conditions. For all patients and controls, parents were present during all the blood drawing and no white coats were worn in the presence of the subject; the subjects stayed in a playroom for 15 minutes before the blood drawing and all phlebotomies were performed by the same nurse, who was particularly experienced with handicapped children. Blood was obtained from the antecubital foci and collected in EDTA-containing tubes, between 8 and 9 am. After centrifugation (4°C, 15 min, 1000 xg), plasma was acidified to pH 4.0 and frozen at −80°C until assayed. Plasma BE concentrations were determined using a previously described radioimmunoassay RIA procedure^58^. Gel filtration on Séphadex G75 was used to isolate a BE-containing fraction. After lyophilization, the fraction was assayed using rabbit anti-serum directed against the C-terminal portion of human BE. The assay is sensitive (limit of detection, 5 pmoles/L) and reproducible (intra- and inter-assay coefficients of variations, 5 and 6% respectively). Results of plasma BE analysis are given only for the samples with acceptable duplicate agreement (57 patients with autism, 103 controls).

### Statistical analyses

Group comparisons of pain reactivity were assessed by χ^2^ test. Relationships between behavioral pain reactivity across the different observational situations were studied by contingency analyses [59]. The Kolmogorov-Smirnov test indicated that plasma BE levels were not normally distributed; thus all statistical analyses were performed using log-transformed BE values. Untransformed means and standard errors for BE levels are given to allow comparisons to previous studies. Group and subgroup comparisons of plasma BE levels were performed using analysis of variance (ANOVA) and two-tailed t-test. Correlations were determined by Spearman or Pearson correlation analyses as appropriate. Based on reported variance in heart rate and plasma BE levels, the study was designed to detect effect sizes of 0.5 or greater.

## Results

### Relationships between descriptive variables and behavioral pain reactivity or BE levels

There were no significant effects of IQ, age, sex, pubertal status and medication status (neuroleptics or anticonvulsants) on behavioral pain reactivity, regardless of the observational situation (parental, caregiver, blood drawing). Significant relationships were also not found between plasma BE levels and IQ, age, sex, or medication status (neuroleptics or anticonvulsants). However, there was a significant effect of puberty on plasma BE log-transformed values (described in a following section).

### Behavioral pain reactivity in the three observational situations

**Figure 1:**
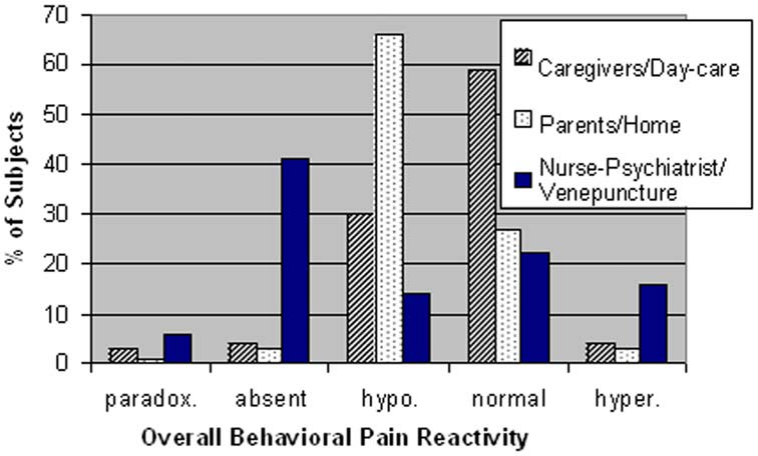
Overall Behavioral Pain Reactivity in Autism According to Rater/Setting. Types/classes of pain reactivity are described in detail in Table 3 and the numerical data are presented in Table 4.

**Figure 2:**
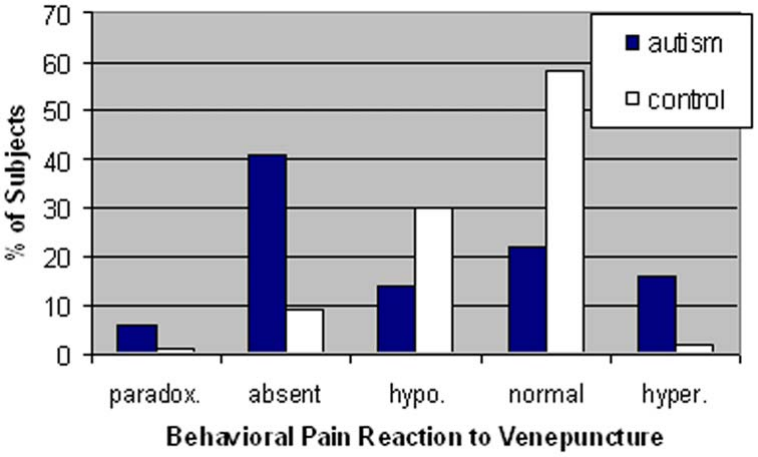
Distribution of types of pain reactivity to venepuncture with nurse and child psychiatrist as observers. Types/class of pain reactivity are described in detail in Table 3 and the numerical data are presented in Table 4.

The distributions of types of behavioral pain reactivity seen in the three observational situations are presented in **Table 4** and **Figure 1**. A large proportion of individuals with autism displayed low/absent pain reactivity according to the parental, caregiver and blood drawing evaluations (68.6%, 34.2%, and 55.6%, respectively). When behavioral pain reactivity to the venepuncture was compared across the autistic and control groups (**Figure 2**), there was a significant and substantial difference in the distribution of pain reactivity responses (χ^2^ (*df* = 4) = 53.68, *P*<0.001). In particular, there were more patients with autism and less controls showing absence of pain reactivity than expected, whereas there were more controls and less patients showing normal pain reactivity than expected (χ^2^ (*df* = 2) = 33.1, *P*<0.0001). Similar results were obtained when comparing drug-free individuals with autistic disorder (n = 47) to normal control subjects (n = 115).

**Table 4:** Evaluation of Behavioral Pain Reactivity in Three Different Observational Situations: Parental, Caregiver, Blood Drawing*.

	Type of Behavioral Pain Reactivity				
	Type I	Type II	Type III	Type IV	Type V
Observational situation	Paradoxical	Absent	Hyporeactivity	Normal	Hyperreactivity
**Home setting/Parental evaluation in individuals with autism (n = 73)**					
Overall pain reactivity	1 (1.4%)	2 (2.8%)	48 (65.8%)	20 (27.4%)	2 (2.8%)
Reaction to burning self	3 (4.1%)	0 (0%)	11 (15%)	57 (78.1%)	2 (2.7%)
Reaction to internal pain (tooth pain, ear infection, etc.)	0 (0%)	2 (2.8%)	34 (47.2%)	33 (45.8%)	3 (4.2%)
Reaction to other pain (accidents, banging self, etc.)	1 (1.4%)	1 (1.4%)	46 (64.8%)	19 (26.8%)	4 (5.6%)
**Day-care/Caregiver evaluation of individuals with autism (n = 73)**					
Overall pain reactivity	2 (2.7%)	3 (4.1%)	22 (30.1%)	43 (58.9%)	3 (4.1%)
**Nurse/Psychiatrist evaluation during venepuncture**					
Individuals with autism (n = 63)	4 (6.3%)	26 (41.3%)	9 (14.3%)	14 (22%)	10 (15.9%)
Normal controls (n = 115)	1 (0.9%)	10 (8.7%)	35 (30.4%)	67 (58.3%)	2 (1.7%)

* Data are given as number of individuals (and % of group) assigned to each class of pain reactivity

However, it is noteworthy that 22% individuals with autism displayed normal pain reactivity to the venepuncture, and 15.9% showed hyperreactivity. In addition, 60.3% of patients with autism (and none of the normal controls) displayed certain autism-associated behaviors immediately after the venepuncture (SIB, aggressive behaviors directed against others, stereotyped behaviors and social withdrawal were observed in 9.5%, 23.8%, 34.9%, and 38.1% of patients, respectively). Similar behaviors following painful stimuli were described by parents and caregivers (SIB, aggressive behaviors directed against others, stereotyped behaviors, social withdrawal). Parents and caregivers also reported that 9 out of the 22 verbal individuals with autism expressed verbal complaints following a painful stimulus, but were unable to locate correctly the painful area.

A comparison using contingency analysis of the parental and caregiver evaluations of overall behavioral pain reactivity type showed no significant relationship between the ratings obtained in the two situations. Similarly, no significant relationship was found between the nurse/psychiatrist ratings during the blood drawing situation and the parental or caregiver evaluations for overall behavioral pain reactivity.

### Heart rate response to venepuncture

Despite the absence of behavioral pain reactivity to the blood drawing observed in many subjects of the autistic group (41.3%), patients with autism displayed robust physiological responses to the venepuncture with a significant increase in heart rate (reactional tachycardia). Furthermore, patients with autism displayed a significantly greater heart rate response to the venepuncture than controls, and had also a significantly higher heart rate before and after the venepuncture than controls (**Table 5**).

**Table 5:** Heart Rates measured during Blood Drawing Procedure.

Heart Rate*	Patients With Autism (n = 63)	Normal Controls (n = 115)	Test of Significance**
Heart rate immediately prior to venepuncture	93.2±2.2	83.3±1.1	*t*_91_ = 4.12, *P*<0.001
Heart rate immediately after venepuncture	99.6±2.5	84.6±1.2	*t*_92_ = 5.41, *P*<0.001
Heart rate response to venepuncture***	6.4±2.5	1.3±0.8	*t*_72_ = 2.0, *P*<0.05

* Heart rate values (number of beats per minute) expressed as mean±SEM.

** Group mean comparison using a two-tailed unequal variance t-tests.

*** Heart rate response to venepuncture (calculated as the heart rate taken a few seconds after the venepuncture minus heart rate a few seconds before the venepuncture) was significant in the group of patients with autism (*t*_62_ = 2.62, *P*<0.05, matched-pairs t-test) and was not significant in the control group (*t*_114_ = 1.54, *P*>0.05, matched-pairs t-test).

### Relationships between diagnosis, autism severity and plasma β-endorphin (BE) levels

**Figure 3:**
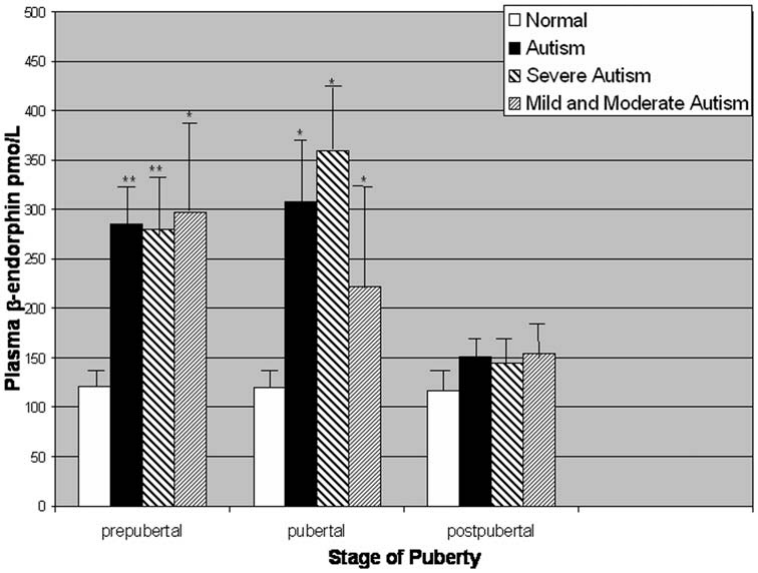
Group mean plasma β-endorphin concentrations. Error bars represent SEM. Asterisks indicate significant differences within pubertal subgroups between normal controls and patients with autism: *P<0.01,**P<0.001. Among the total group of individuals with autism, both pre-pubertal and pubertal individuals had significantly higher β-endorphin levels than post-pubertal individuals (*P*<0.01).

A three-way ANOVA including group (patients, controls), puberty (pre-pubertal, pubertal, post-pubertal) and sex factors showed a significant effect of group (F_1,146_ = 37.22, *P*<0.001), puberty (F_2,146_ = 6.04, *P*<0.01) and group interacting with puberty (F_2,146_ = 5.80, *P*<0.01) (**Figure 3**). Patients with autism (n = 57) had significantly higher plasma BE levels (mean±SE, 231.5±24.1, versus 119.5±5.3 pmol/L) than controls (n = 103). An ANOVA including puberty and autism severity showed a significant effect of autism severity on plasma BE levels (F_2,151_ = 11.05, *P*<0.001): mean plasma BE levels observed in individuals with “severe” autism (244.2±30.7, n = 39) were higher than levels in individuals with “mild” to “moderate” autism (214.4±41.6, n = 18) which were higher than in normal controls (119.5±5.3, n = 103). Plasma BE levels were significantly higher in the severe autism subgroup (*t*_140_ = 4.05, *P*<0.01) and in the mild/moderate autism subgroup (*t*_119_ = 3.01, *P*<0.01) compared to the control group. Considering that IQ scores were significantly negatively correlated with autism severity (Spearman *r* = −0.24, *P*<0.05), group mean plasma BE levels in the severe and mild/moderate subgroups were also compared using analysis of covariance (ANCOVA), with IQ entered as a covariable. Similar, significant results were obtained using the covariant analysis.

### Relationships between plasma BE levels and pain reactivity

Using contingency analyses, no significant relationships were observed between plasma BE levels and behavioral pain reactivity assessed in any of the three observational situations (caregiver, parental, blood drawing) for patients with autism. Pain reactivity of controls during the blood drawing was also not significantly related to plasma BE levels. Levels of plasma BE were positively correlated with heart rate before (r = 0.28, *P*<0.05) and after the venepuncture (r = 0.29, *P*<0.05) in individuals with autistic disorder.

## Discussion

We clearly show across the three observational situations that abnormal behavioral responses to painful stimuli were highly prevalent in individuals with autism of low to moderate functioning. In general, there was a shift to hypo-reactive or absent pain reactions in the autism group. Although this pattern of observed behavior is consistent with a number of previous studies, most prior reports did not distinguish pain reactivity from pain sensitivity. As will be discussed, it is critical to keep this distinction in mind and not to conclude that absence of behavioral pain reactivity means absence of pain sensitivity. Indeed, one of our major findings was that despite many subjects of the autism group (41.3%) displaying absence of behavioral pain reactivity to the venepuncture, individuals with autism showed a robust physiological response with a significant reactional tachycardia to the venepuncture. This suggests that individuals with autism experienced the noxious stress of the venepuncture and that their apparent behavioral analgesia could be related more to a different mode of pain response than to a real endogenous analgesia. Moreover, they displayed significantly higher stress responses to the general blood drawing situation than controls (with higher mean values of heart rate just before or after venepuncture, and higher tachycardia to the venepuncture).

The greater heart rate response to venepuncture in the autistic group compared to the control group may be a consequence of several factors operating singly or in concert. Greater amounts of situational anxiety in the autistic group are consistent with their higher baseline heart rate and would be expected to increase pain sensitivity [60]. Autistic individuals may also perceive the needle insertion as more invasive and more novel than control individuals. In addition, they may be less able to regulate their emotional response to pain or to express their noxious stress through verbal and non-verbal communication, and also may be less capable of invoking adaptive strategies such as deliberate self-distracting, exhibiting praiseworthy stoicism, assuring oneself of the time-limited nature of the process, or imagining comforting that will be shortly forthcoming.

However, a key question should be addressed: does the reactional tachycardia to the venepuncture represent a valid physiological indicator of pain? Indeed this reactional tachycardia could rather reflect the stress of the autistic child of being held and immobilized during the blood drawing. However, aggressively restrictive measures were avoided and more specifically the reactional tachycardia was calculated as the heart rate taken immediately after the venepuncture minus the heart rate immediately before the venepuncture when the child was already being hold. Alternatively, the perception of the needle entering the body or the sight of drawn blood could have been stressful. Stress arising from the former possibility can not be ruled out; however, the latter possibility can be ruled out because the nurse used a Vacutainer system in such a way the child could not see the blood.

In addition to the physiological response to the venepuncture, behavioral changes following the venepuncture or other painful stimuli occurring at home and day hospital (SIB, aggressive behaviors, stereotyped behaviors, social withdrawal) also suggest that children with autism perceive pain, but do not express it in the same way as normal children. Furthermore, our results indicated that substantial proportions of individuals with autism did not display low/absent overall pain reactivity according to the parental, caregiver and blood drawing evaluations. More specifically, a majority (78%) of the autistic individuals actually displayed a normal behavioral reactivity to burning; this underlines the importance of distinguishing different types of painful stimuli. Also, our results indicate that 22% individuals with autism displayed normal behavioral pain reactivity to the venepuncture and 15.9% displayed hyperreactivity which is in agreement with Nader et al.'s important study [61] finding on average (mean values) normal behavioral responses or even excessive facial pain reactivity (assessed by detailed coding of videotapes using the Child Facial Coding System [62]) to the venepuncture in 21 children with autism compared to 22 non-impaired children. Taken together, these observations constitute a clear challenge to theories of reduced pain sensitivity in autism.

Given our results, we can hypothesize that painful stimuli provoke physical and psychic stress and that in autism this stress can be manifested by physiological responses and expressed through autistic behaviors. The different mode of pain expression in autism may be related to 1) verbal communication impairments, 2) deficits in non-verbal communication and body image problems (difficulty locating the painful area), or 3) other cognitive problems such as: (a) difficulty in establishing cause-effect relationships between the pain sensation and the stimulus causing the pain, (b) problems discriminating, representing and identifying sensations and emotions which involves abstraction and symbolisation capacities (the perception of pain integrates sensorial, emotional, and cognitive factors [63]), (c) problems of learning socially appropriate responses to pain, as highlighted by Glover's definition of pain in which sensation, suffering, and learning are integral parts [64].

The notion of non-perception of pain (or decreased pain sensitivity) in autism is similar to ideas once proposed for infants [65]. The denial of infant pain was based on the idea of neurological and psychological immaturity and a lack of knowledge of the particular pain semiology related to the absence of verbal language. There are also striking parallels between prior pain-related research in autism and in schizophrenia. In both autism and schizophrenia [66]–[67], pain insensitivity was widely accepted without being empirically established through objective methods and opioid theories were posited. However, certain studies have reported distorted perception of pain (atypical pain including hyperreactivity to pain) related to social communication impairments and a dissociation between decreased behavioral and increased physiological responses in schizophrenia [66]–[71], which suggests, as in our study, that apparent pain insensitivity in schizophrenia is related to a different mode of pain expression and response. Overall, the patterns of response to pain that we observed, and the offered interpretations, appear largely consistent with prior studies of pain in a range of relevant groups, including young children, the severely neurological impaired, psychiatric disorders, mentally retarded and the developmental delayed [41]–[51]. In these groups, it appears likely that fundamental aspects of pain sensitivity are intact and that altered responses to painful stimuli are usually due to differences in experiencing and difficulties in communicating the experience of pain. Autism appears to present a special case where various factors seen operating in the groups listed above can combine to lead to marked problems in this area. Thus, difficulties in expressing pain in autistic individuals could arise from general deficits in verbal and non-verbal communication and from differences in the higher level processing and consideration of pain.

Another potentially important finding was that group patterns of pain reactivity differed depending upon the observational situation. The results underline the key role of the situation and the observers in evaluations of pain reactivity. Our findings are in agreement with Nader's study [61] showing different results of behavioral pain reactivity according to the observational situation. Thus, the rating obtained by clinicians using the Child Facial Coding System during the blood drawing procedure was not correlated with either parental estimates of pain to the venepuncture or parental report of pain sensitivity history [61]. This dependence of pain behavioral response assessment on observer, and the potential influence of the observer/rater and setting on the behavior itself, have been previously examined and discussed [44].The different results between the three observational situations underline the relational aspect that seems to exist in the expression of behavioral pain reactivity. This relational aspect manifests itself (a) in the patient who may modulate his/her behaviors as a function of the environment, and (b) in the observer who can perceive and interpret, and thus score subjectively behavioral pain reactivity. Behavioral pain reactivity involves a communicative dimension with a person who expresses him/herself through a behavior related to a situation, and another person who interprets and reacts to this behavior. It should also be pointed out that the Nader study used a stop-frame and slow-motion video display to assess facial expressions during venpuncture, and actually found increased reactivity to pain [61]. Thus, their results are consistent with the main conclusion of our study that individuals with autism do not have decreased sensitivity to pain.

An additional important finding was the presence of significantly higher plasma BE levels in patients with autism compared to normal controls, an elevation that was associated with autism severity, but not with behavioral pain reactivity. The group elevation in BE is consistent with our and several others' prior studies [29], [32]–[34]. The absence of a group difference in post-pubertal subjects is consistent with the blood drawing being less stressful for the older children and young adults with autism, although the reasons for this are unclear. The absence of an association between plasma BE and behavioral pain reactivity is consistent with the fact that during stress exposure peripheral BE is co-released with adrenocoticotrophin hormone (ACTH) from the pituitary and does not cross the blood-brain barrier. When measured in plasma, BE should be considered a stress hormone [72]–[74] and not an indicator of central opioid functioning. The positive correlations observed between plasma BE levels and pre- or post-venepuncture heart rate also tend to support this view. As with the observed increased pre- and post venepuncture heart rates, the increased group mean BE level in autism (which probably reflects an increased activation of the hypothalamic-pituitary-adrenal axis) appears to be associated with the stress of the blood drawing setting and not with the observed pain-related behavior.

The apparent absence of real endogenous analgesia, as well as the absence of clear benefits of opiate antagonist therapies and the inconsistent results of studies measuring central opioid levels in autism, all tend to weigh against opioid theories of autism. However, our data do not allow us to definitively rule out the theory of an excessive central BE activity in autism. Assessment of possible central opioid alterations will require additional CSF studies, neuroendocrine challenge studies, brain imaging or post-mortem analyses. The increased plasma BE levels and heart rates observed in individuals with autism during the blood-drawing situation appear to reflect enhanced biological and physiological stress responses which are dissociated from observable emotional and behavioral reactions. Further research in larger groups is urgently needed to characterize more fully pain sensitivity and reactivity in autism, to understand the role of anxiety and stress response system functioning in pain-related behavior, and to improve the communication of distress in individuals with autism. An increased awareness of the importance of stressful and painful events in the care of individuals with autism is recommended. In particular, a greater vigilance needs to be exercised in the care of children with autism with regard to their exposure to painful stimuli and their difficulty in expressing their behavioral response to pain, knowing that altered or reduced behavioral pain reactivity does not mean reduced pain sensitivity. We hope the present study will contribute to a greater appreciation of the varieties of pain expression in autism and will lead to a reconsideration of the prevalent assumption that children with autism are insensitive or indifferent to pain.
